# Supplementary material for: Sleep quality of college students in Fujian and its influencing factors: A cross-sectional study
Source: PLoS One. 2025 Apr 16;20(4):e0319347. doi: 10.1371/journal.pone.0319347 (PMC12002490; doi:10.1371/journal.pone.0319347)
Supplement: S1 Table — (DOCX) [file pone.0319347.s001.docx]

**S1 Table.** The differences between PSQI general and component scores among ages（$\overline{\text{x}}\pm\text{s}$）

| **Indices** | ＜**20years（n=315）** | ≥**20years（n=656）** | ***t*** | ***P*** |
| --- | --- | --- | --- | --- |
| PSQI general scores | 4.40±3.32 | 4.68±2.96 | -1.398 | 0.163 |
| PSQI component scores |  |  |  |  |
| Subjective sleep quality | 0.99±0.77 | 1.01±0.70 | -0.142 | 0.887 |
| Sleep latency | 1.13±1.17 | 1.25±1.15 | -2.158 | 0.031^*^ |
| Sleep duration | 0.71±0.85 | 0.65±0.83 | 1.337 | 0.182 |
| Habitual sleep efficiency | 0.45±0.82 | 0.42±0.76 | 0.170 | 0.865 |
| Sleep disturbances | 0.78±0.65 | 0.91±0.60 | -3.412 | ＜0.001^*^ |
| Use of sleep medications | 0.08±0.38 | 0.10±0.44 | -0.953 | 0.341 |
| Daytime dysfunction | 0.25±0.63 | 0.33±0.73 | -1.040 | 0.298 |

* indicate *p*＜0.05
