# Supplementary material for: Sleep quality of college students in Fujian and its influencing factors: A cross-sectional study
Source: PLoS One. 2025 Apr 16;20(4):e0319347. doi: 10.1371/journal.pone.0319347 (PMC12002490; doi:10.1371/journal.pone.0319347)
Supplement: S2 Table — (DOCX) [file pone.0319347.s002.docx]

**S2 Table.** The differences between PSQI general and component scores among sex（$\overline{\text{x}}\pm\text{s}$）

| **Indices** | **Male**  **（n=541）** | **Female**  **（n=430）** | ***t*** | ***P*** |
| --- | --- | --- | --- | --- |
| PSQI general scores | 4.37±3.22 | 4.59±3.14 | -1.006 | 0.315 |
| PSQI component scores |  |  |  |  |
| Subjective sleep quality | 0.95±0.75 | 1.03±0.74 | -2.516 | 0.012^*^ |
| Sleep latency | 1.08±1.17 | 1.23±1.16 | -2.728 | 0.007^*^ |
| Sleep duration | 0.80±0.87 | 0.63±0.83 | 2.143 | 0.032^*^ |
| Habitual sleep efficiency | 0.39±0.78 | 0.46±0.81 | -1.204 | 0.229 |
| Sleep disturbances | 0.70±0.65 | 0.90±0.62 | -5.036 | ＜0.001^*^ |
| Use of sleep medications | 0.14±0.50 | 0.07±0.35 | 1.953 | 0.051 |
| Daytime dysfunction | 0.32±0.69 | 0.27±0.67 | 2.068 | 0.039^*^ |

* indicate *p*＜0.05
