# Supplementary material for: Sleep quality of college students in Fujian and its influencing factors: A cross-sectional study
Source: PLoS One. 2025 Apr 16;20(4):e0319347. doi: 10.1371/journal.pone.0319347 (PMC12002490; doi:10.1371/journal.pone.0319347)
Supplement: S3 Table — (DOCX) [file pone.0319347.s003.docx]

**S3 Table.** The differences between PSQI general and component scores among education（$\overline{\text{x}}\pm\text{s}$）

| **Indices** | **College**  **((n=515)** | **Undergraduate**  **（n=416）** | **Postgraduate**  **（n=40）** | ***F*** | ***P*** |
| --- | --- | --- | --- | --- | --- |
| ^a^ PSQI general scores | 4.76±3.28 | 4.24±3.01 | 4.47±3.17 | 3.088 | 0.046^*^ |
| PSQI component scores |  |  |  |  |  |
| Subjective sleep quality | 1.01±0.72 | 1.01±0.76 | 0.88±0.82 | 0.774 | 0.461 |
| Sleep latency | 1.24±1.29 | 1.09±0.99 | 1.30±1.14 | 0.101 | 0.904 |
| Sleep duration | 0.76±0.86 | 0.59±0.80 | 0.68±0.97 | 0.076 | 0.927 |
| Habitual sleep efficiency | 0.43±0.78 | 0.44±0.82 | 0.43±0.81 | 0.185 | 0.831 |
| Sleep disturbances | 0.81±0.62 | 0.88±0.65 | 0.85±0.66 | 0.828 | 0.437 |
| Use of sleep medications | 0.06±0.32 | 0.12±0.47 | 0.15±0.58 | 2.650 | 0.071 |
| Daytime dysfunction | 0.44±0.83 | 0.10±0.36 | 0.20±0.61 | 1.918 | 0.148 |

* indicate *p*＜0.05; a indicate pairwise comparison was calculated using the LSD test.
