# Supplementary material for: Sleep quality of college students in Fujian and its influencing factors: A cross-sectional study
Source: PLoS One. 2025 Apr 16;20(4):e0319347. doi: 10.1371/journal.pone.0319347 (PMC12002490; doi:10.1371/journal.pone.0319347)
Supplement: S4 Table — (DOCX) [file pone.0319347.s004.docx]

**S4 Table.** The differences between PSQI general and component scores among major（$\overline{\text{x}}\pm\text{s}$）

| **Indices** | Medical  **（n=412）** | **Non-medical**  **（n=559）** | ***t*** | ***P*** |
| --- | --- | --- | --- | --- |
| PSQI general scores | 4.76±3.36 | 4.35±3.01 | -1.989 | 0.047^*^ |
| PSQI component scores |  |  |  |  |
| Subjective sleep quality | 0.99±0.72 | 1.01±0.76 | 1.583 | 0.114 |
| Sleep latency | 1.23±1.30 | 1.15±1.05 | 1.477 | 0.140 |
| Sleep duration | 0.79±0.86 | 0.61±0.83 | 1.087 | 0.277 |
| Habitual sleep efficiency | 0.41±0.77 | 0.46±0.82 | 0.028 | 0.978 |
| Sleep disturbances | 0.78±0.63 | 0.88±0.64 | 1.921 | 0.055 |
| Use of sleep medications | 0.06±0.32 | 0.12±0.46 | 2.197 | 0.028^*^ |
| Daytime dysfunction | 0.50±0.88 | 0.12±0.41 | 1.892 | 0.059 |

* indicate p＜0.05
