# Supplementary material for: Sleep quality of college students in Fujian and its influencing factors: A cross-sectional study
Source: PLoS One. 2025 Apr 16;20(4):e0319347. doi: 10.1371/journal.pone.0319347 (PMC12002490; doi:10.1371/journal.pone.0319347)
Supplement: S5 Table — (DOCX) [file pone.0319347.s005.docx]

**S5 Table.** Tolerance and variance inflation factors for different variables

| **Variable** | **Tolerence** | **VIF** |
| --- | --- | --- |
| Age（year） | 0.964 | 1.037 |
| Sex | 0.878 | 1.140 |
| Education | 0.632 | 1.583 |
| Major | 0.659 | 1.518 |
| Monthly living expenses (yuan) | 0.475 | 0.845 |
| Tobacco | 0.055 | 0.769 |
| Alcohol | 0.600 | 0.816 |
| Beverage | 0.176 | 0.949 |
| Eating habits | 0.036 | 0.957 |
| Electronic product addiction | 0.000 | 0.939 |
| Personal history of acute illness | 0.000 | 0.904 |
| Personal history of chronic disease | 0.055 | 0.922 |
| Parental sleep problems | 0.021 | 0.969 |
